# Multiple evanescent white dot syndrome (MEWDS) following inactivated COVID-19 vaccination (Sinovac-CoronaVac)

**DOI:** 10.5935/0004-2749.20220070

**Published:** 2025-08-21

**Authors:** Kenzo S. Tomishige, Eduardo A. Novais, Luciana P. dos S. Finamor, Heloísa M. do Nascimento, Rubens Belfort Jr.

**Affiliations:** 1 Instituto Paulista de Estudos e Pesquisa em Oftalmologia, Instituto da Visão, São Paulo, SP, Brazil; 2 Department of Ophthalmology, Universidade Federal de São Paulo, São Paulo, SP, Brazil; 3 Centro Oftalmológico Città, Rio de Janeiro, RJ, Brazil

**Keywords:** White dot syndrome/diagnosis, COVID-19, Coronavirus infection, Vaccine, Vaccination, Adrenal cortex hormones, Múltiplos pontos brancos/diagnóstico, COVID-19, Infecção por coronavirus, Vacina, Vacinação, Corticosteroides

## Abstract

A 38-year-old woman presented with photopsias and progressive but painless loss of vision in her right eye. Of note, she had received the first dose of inactivated COVID-19 vaccine (Sinovac/China National Pharmaceutical Group) 2 weeks prior to the onset of symptoms. Ophthalmic evaluation revealed a wreath-like foveal pattern and multiple gray-white dots throughout the posterior pole associated with discrete vitreous inflammatory reaction. Multimodal imaging analysis confirmed a diagnosis of multiple evanescent white dot syndrome. The patient underwent treatment with corticosteroids and, over the following weeks, her visual acuity improved to standard pattern.

## Abstract (pt)

Paciente feminina de 38 anos com queixa de fotopsias e perda visual indolor em seu olho direito 2 semanas após ter recebido a primeira dose de vacina de vírus inativo para COVID-19 (Sinovac/China National Pharmaceutical Group). Ao exame Oftalmológico, apresentava padrão granular macular, múltiplas lesões cinza-esbranquiçadas no polo posterior, associado à reação vítrea inflamatória. O diagnóstico de múltiplos pontos brancos evanescentes foi realizado após análise multimodal. A paciente foi tratada com corticosteroides sistêmico e evoluiu com melhora da acuidade visual e do quadro clínico.

## INTRODUCTION

Multiple evanescent white dot syndrome (MEWDS) is a rare diagnosis within the group of white dot/white spot syndromes (WDS)^(1)^. MEWDS is typically unilateral and occurs in healthy people, mainly young and myopic women. Notably, the disease is associated with a viral prodrome in about 30% of cases^(1)^. The classic clinical presentation of MEWDS is low visual acuity without pain accompanied by transient visual disturbances such as blurred vision, photopsias, and blind spot enlargement. On ophthalmic examination, the biomicroscopic findings are usually discrete; in contrast, on fundoscopic examination, the most common findings are multifocal gray-white lesions that affect the posterior pole at the outer retinal level and the retinal pigment epithelium (RPE)^(1)^. Further examinations, such as optical coherence tomography (OCT), fundus autofluorescence (FAF), and fluorescein angiography (FA), can support diagnosis and the follow-up of patients^(1)^.

Since the onset of the COVID-19 pandemic, distinct ophthalmologic findings have been reported after SARS-CoV-2 infection and COVID-19 vaccination^(2-4)^. MEWDS has been previously reported in patients who received the BNT162b2 mRNA vaccine^(3)^. Here we report the first case of MEWDS following inactivated COVID-19 vaccination.

## CASE REPORT

We present a case of a 38-year-old white female patient with photopsias and painless low visual acuity in the right eye lasting for 2 weeks. She had received the first dose of inactivated COVID-19 vaccination (Sinovac-CoronaVac, Sinovac/China National Pharmaceutical Group) 3 weeks before presentation at our hospital and reported symptom onset 7 days after vaccination.

**Figure 1:**
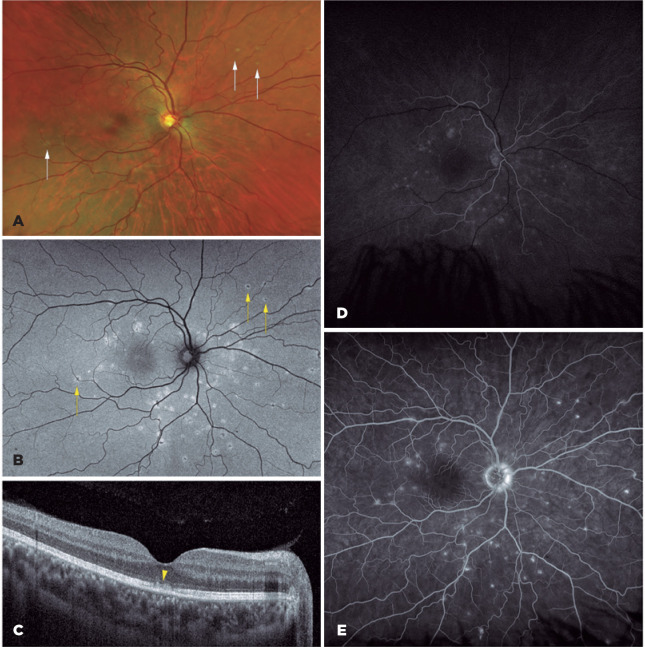
Baseline multimodal imaging analysis of the right eye. (A) The multicolor fundus image shows altered macular reflex and retinal pigment epithelium atrophy with multiple gray-white dots at the macula and midperiphery (white arrows). (B) Fundus autofluorescence indicates widespread multiple hyperautofluorescent lesions in the outer retina (yellow arrows). (C) Spectral domain optical coherence tomography reveals a fragmented ellipsoid zone and irregular reflectivity of the retinal external limiting membrane at the center of the fovea (yellow arrowhead). (D and E) Widefield fluorescein angiography shows hyperfluorescent dots spread throughout the retina during the venous (D) and arteriovenous (E) phases, as well as leakage at the nasal section of the optic nerve.

Ophthalmic examination revealed normal pupilar reflexes and best-corrected visual acuity of 20/400 in the right eye (OD) and 20/20 in the left eye (OS). Biomicroscopy showed 1+ anterior chamber inflammatory reaction and 1+ vitreous cells in the OD and normal results in the OS. Intraocular pressure (IOP) was normal in both eyes (OU). Fundoscopy showed a pink optic disc with sharp margins, altered macular reflex, normal vessels with RPE atrophy, and multiple gray-white dots in the posterior pole of the OD; the OS was normal. The patient’s past ophthalmic and medical histories were otherwise unremarkable. The fundus image showed white spots (Figure 1A) presenting as hyperautofluorescent dots on the fundus autofluorescence (FAF) (Figure 1B). Macular OCT (Figure. 1C) revealed thickening of the retina’s outermost layers plus foveal RPE uniformity loss. The FA (Figure 1D and 1E) presented a wreath-like pattern, matching the lesions at the level of the RPE.

**Figure 2:**
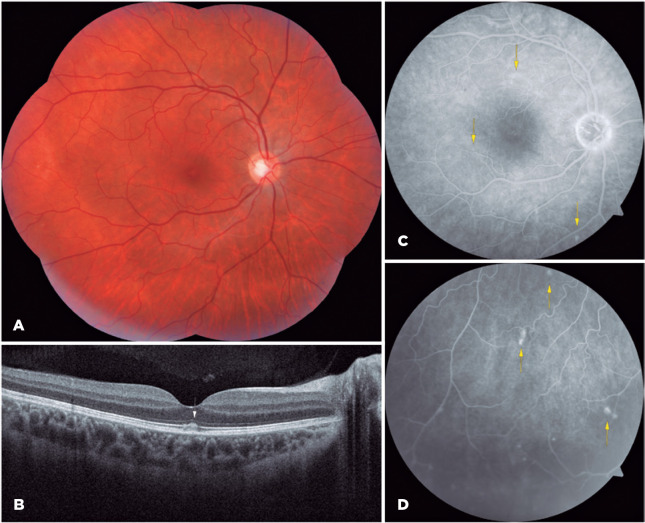
Multimodal imaging analysis of the right eye during follow-up. (A) The fundus image of the right eye shows partial regression of the white lesions and foveal changes. (B) Spectral domain optical coherence tomography of the right eye shows improvement of ellipsoid zone reflectivity (white arrow). (C and D) Fluorescein angiography shows persistence of the hyperfluorescent dots (yellow arrows) with decreased leakage at the nasal section of the optic nerve.

The patient was treated with 80-mg/day oral prednisone for 1 week, after which the dose was tapered. After 4 weeks, there was improvement in visual acuity (20/20 OU). The fundus image showed regression of the white retinal dots. (Figure 2A), OCT showed significant improvement in the ellipsoid zone (Figure 2B), and FA showed persistence of hyperfluorescence with decreased optical nerve leakage (Figure 2C and D).

## DISCUSSION

As various COVID-19 vaccines are developed and administered around the world, it is expected to see different outcomes concerning immunization efficacy and side effects^(5)^. Concerns about adverse post-vaccination events, especially the appearance of uveitis, are long-standing and currently increasing^(6)^.

This report describes a young, previously healthy woman who developed photopsias and low visual acuity 1 week following SARS-CoV-2 vaccination (inactivated Sinovac-CoronaVac vaccine, Sinovac/China National Pharmaceutical Group). The findings of multimodal images were consistent with MEWDS. While this disorder is thought to have a benign prognosis with spontaneous resolution^(1)^, due to the patient’s impaired vision and complaints, she was treated with oral steroids. With treatment, the symptoms and ophthalmologic findings resolved 4 weeks after initial presentation. Although as many as 30% of MEWDS patients report a viral prodrome^(1)^, this patient curiously did not report systemic symptoms.

Notably, there is a prior report of MEWDS following vaccination for influenza, a vaccine that, like the vaccine in the present case, uses inactivated virus^(7)^. Furthermore, a recent study found two cases of MEWDS following the second doses of the BNT162b2 mRNA SARS-CoV-2 vaccine (Pfizer BioNTech), which belongs to a new class of vaccines based on RNA technology^(3)^. Proposed mechanisms of MEWDS following vaccination include molecular mimicry, antigen-specific cell and antibody-mediated hypersensitivity reactions, and/or adjuvant-mediated inflammation^(6)^. As viral particles have been observed in the retinas of patients who died of COVID-19^(8)^, as well as in cerebral astrocytes^(9)^, another hypothesis is greater tropism of the virus in the central nervous system and retina, with the eventual deposition of vaccine antigens in the retinal tissue being the trigger of the disease. Further research on these potential mechanisms is warranted.

With vaccination rapidly increasing worldwide, the possibility of new adverse events-especially in patients with immune-mediated conditions-is of important consideration^(10)^. Besides MEWDS, there have been other notable ocular findings regarding post-inactivated vaccination, such as episcleritis, anterior scleritis, acute macular neuropathy, paracentral acute middle maculopathy, and sub-retinal fluid. These were also reversible events and, at present, no causal relationship has been established^(4)^. In addition, there is no evidence to impose contraindications of vaccines for immunocompromised patients or those with autoimmune diseases. Nevertheless, there is a possibility that vaccination under immunosuppressive therapies could reduce potential responses^(10)^.

While inactivated COVID-19 vaccine may be associated with MEWDS, the possibility of just a temporal association cannot be ruled out. The COVID-19 pandemic is pushing the world’s vaccination numbers to an unprecedented level. As it is possible that we will have an increasing number of ocular adverse events, it is of great importance to describe new findings. For most patients, the benefits of vaccination in the face of a pandemic and its consequences far outweigh the risks of uveitis.
